# Improvements in high-yield detection and localisation of cholesterol in murine brains using water cluster beam secondary ion mass spectrometry

**DOI:** 10.1007/s00216-026-06448-8

**Published:** 2026-03-26

**Authors:** Matija Lagator, Irma Berrueta Razo, Sadia Sheraz, Nicholas P. Lockyer

**Affiliations:** 1 Department of Chemistry, Photon Science Institute, The University of Manchester, Oxford Road, Manchester, M13 9PL UK; 2 Rosalind Franklin Institute, Building R113 Rutherford Appleton Laboratory, Harwell Campus, Didcot, Oxfordshire OX11 0QX UK; 3 Medicines Discovery Catapult, 35 Mereside Alderley Park, Macclesfield, SK10 4ZF UK

**Keywords:** Mass spectrometry imaging, Secondary ion mass spectrometry, Cholesterol, GCIB, Water clusters, Brain tissue, Matrix effects

## Abstract

Cholesterol is a neutral lipid widely implicated in neurological and other physiological processes. However, it is difficult to detect and precisely localise in situ using mass spectrometry imaging techniques due to low sensitivity and matrix suppression by co-localised lipids. Here, we present the application of large water gas cluster ion beams (GCIBs) for cholesterol analysis and localisation in murine brains using secondary ion mass spectrometry (SIMS). Optimal GCIB parameters were established through analysis of cholesterol standards. These clusters were then used to analyse whole brain sections with minimal sample preparation. Molecular images obtained with water clusters show that cholesterol is distributed across the whole brain, with variations in intensity. In addition, large water clusters were shown to simultaneously maximise secondary ion yields while reducing matrix effects for in situ cholesterol analysis. Depth profiling further revealed that different brain regions produced varying sputter yields. This study demonstrates that large water clusters are very well suited for cholesterol analysis in murine brains. Because this approach requires no derivatisation or matrix addition, it is likely to make analysis faster, simpler, and less prone to matrix effects.

**Graphical Abstract:**

**Figure:**
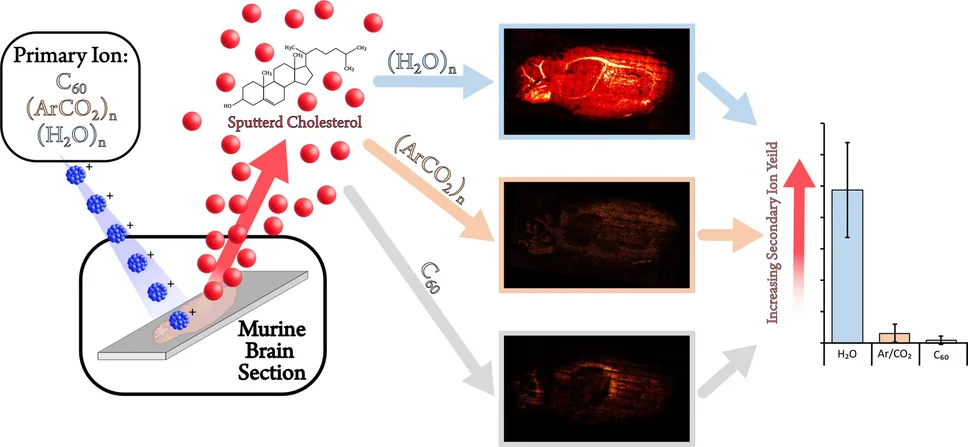

**Supplementary Information:**

The online version contains supplementary material available at https://doi.org/10.1007/s00216-026-06448-8.

## Introduction

Cholesterol has a vital role as a structural component in cell membranes and in lipid metabolism, among other crucial biological functions [1]. It is an essential molecular component in the brain, where the concentration of cholesterol is around 23 mg/g [2, 3] and around 70–80% of this amount is contained in myelin. Cholesterol is responsible for the proper functioning of the myelin sheaths [4, 5] which act as exon insulators and are essential for rapid electrical signal transduction. Axons are mostly located in white brain regions and as a consequence cholesterol strongly localises in these regions [6]. Cholesterol is also a key component of lipoproteins in membrane lipid rafts used for cell signalling [7]. It interacts with other membrane phospholipids thus affecting the physical and biological characteristics of the membrane [8], in addition to performing a central role in a range of signal transduction mechanisms [9]. With this wide area of cellular functions, it follows that defects in cholesterol mechanisms also result in a variety of pathologies. It has been found that cholesterol is implicated in both Parkinson’s disease [10, 11] and Alzheimer’s disease (AD) [12–14].

With this range of functions and varied distribution of cholesterol in brain tissue, there is a need for more precise, accurate, and efficient methodology for molecular localisation. Mass spectrometry has the capability for in situ compound detection while providing localisation information. Through multi-dimensional mass spectrometry analysis, it was possible to localise approximately 200 sterols in a mouse brain sample [15]. In this experiment, the different mouse brain regions were dissected by hand which greatly reduced the overall amount of spatial information obtained from the experiment. Mass spectrometry imaging (MSI) can provide in situ information about localisation of cholesterol molecules in biological samples with little or no need for sample preparation. However, molecular imaging of cholesterol has presented difficulties to date, as various mass spectrometry techniques have not been optimised for this type of analysis [16, 17]. This is compounded by the low ionisation efficiency of cholesterol molecules. Methodologies have been developed to circumvent this issue of low ionisation probability. For instance, Angelini et al. detected cholesterol in a mouse brain section by applying a derivatisation approach with MALDI-MSI (matrix-assisted laser desorption/ionisation-MSI) [18]. Cholesterol can also be detected with other MSI techniques such as time-of-flight (ToF) SIMS, which offers the advantage of non-targeted detection of molecules directly from the sample’s surface with no need for matrix application or sample derivatisation. Additionally, SIMS provides comparatively higher spatial resolution compared to other MSI methodologies such as MALDI or DESI (desorption electrospray ionisation) [19]. The resolution of ToF-SIMS is routinely in the < 1–10 μm range for molecular species, depending on the primary ion beam applied [20]. MALDI and DESI can reach resolutions of 1.4 μm [21] and 10 μm [22] respectively, but these spatial resolutions are not easily achievable. Different MS techniques have been shown to poorly ionise cholesterol and methods such as MS/MS, high resolution MS, or derivatisation for cholesterol detection [16, 17, 23, 24] are often employed. ToF-SIMS, especially when applied with water clusters, is well suited for ionisation and analysis of lipids, including cholesterol [23, 25, 26]. However, some potential disadvantages that the MSI field as a whole faces are low ionisation yields, matrix effects, molecular migration, surface damage, and molecular fragmentation [20].

Polyatomic primary ion (PI) beams (SF_6_ [27] and C_60_ [28]) and gas cluster ion beams (GCIBs) (using Ar [29], CO_2_ [30], and H_2_O [31]) have been developed to enhance the performance of ToF-SIMS for molecular determination. Compared to monoatomic PIs, analysis with gas clusters has inferior spatial resolution but reduces surface damage and lowers molecular fragmentation. Past experiments performed on cholesterol focused on the analysis of fragments produced by mono- or polyatomic primary ion beams. In 2008, Piehowski et al. showed that by applying MS/MS methodology with C_60_ or indium primary ion, it is possible to detect diagnostic fragments of cholesterol including at *m/z *147 [32]. Metal coating has also been applied to reduce fragmentation levels. Using this metal-assisted ToF-SIMS methodology, it was possible to localise cholesterol on cell imprints [33], on Ag-covered cells [34], in rat kidney section [35], and in the nervous tissue of *Lymnaea stagnalis* [36]. Another related concern when performing ToF-SIMS analysis is the migration of chemical species as a result of sample preparation and exposure to high vacuum within the ToF-SIMS instrument. It has been shown that it is possible to mitigate molecular migration, as well as sublimation, by analysing the sample under cryogenic conditions [6, 37–39]. This was substantiated by the visualisation of cholesterol in a rat cerebral cortex [37] and through depth profiling of rat brain sections [38].

ToF-SIMS analysis with GCIBs has been demonstrated to reduce the matrix effect, while increasing secondary ion yields [31, 40–42]. Matrix effects arise from changes in the secondary ion formation probability of compounds caused by their chemical or physical environment [41, 42]. The most commonly used GCIB gases are argon and carbon dioxide, or a mixture of the two. More recently, water has also been used as it provides higher ion yields and further reductions in matrix effects in model samples [31, 40]. The hypothesised mechanism for these improvements is based on the presence of hydrogen atoms in the primary ions and their ability to transfer to sputtered species. A higher rate of proton transfer leads to the formation of more secondary ions and thus an increase in secondary ion yields. Furthermore, by providing a large number of protons for secondary ion formation, water clusters reduce the extent to which sputtered chemical species need to ‘compete’ for hydrogen atoms [42]. This increases ionisation efficiency more uniformly across different chemical environments, thereby reducing matrix effects.

Another beneficial aspect of the water clusters is that they can be relatively easily altered in order to change the nature of the primary ions. This is observed through the changing of the carrier gas used to transport the clusters down the primary ion column [26, 43, 44]. Additionally, water added to the surface of the sample in the analysis chamber can interact with HCl that has been doped into the argon clusters [41]. This too results in increased yields as a consequence of the chemically reactive nature of the beam.

In this study, we apply CO_2_-doped water clusters to the analysis of cholesterol in mouse and rat brains. This approach demonstrates increased ionisation yields and reduced matrix effects in brain tissue, resulting in improved mass spectrometry imaging performance. Water clusters also proved useful for assessing relative differences in sputter yields across distinct brain regions. This has important implications for both visualisation and quantification, as variations in sputter rate must be considered in future analyses. Moreover, cholesterol has been particularly difficult to analyse using other mass spectrometry techniques. By eliminating the need for derivatisation or matrix application, the use of large water clusters provides a simpler and faster methodology for analysing and localising cholesterol in biological samples, while maintaining high secondary ion yields and spatial resolution. This work introduces a novel application of doped water clusters, specifically CO_2_ doping, to enhance both ionisation and sputtering behaviour in complex biological tissues. While water cluster beams have been explored previously, their modification through gas doping and application to sterol imaging represents a new direction for ToF-SIMS. This presents a robust approach for imaging molecules that are otherwise difficult to ionise, with minimal sample preparation. The improved detection of sterols and other low-yield biomolecules further demonstrates the potential of doped water clusters to deliver more accurate and quantitative mass spectrometry imaging.

## Methods

### Cholesterol standards - preparation

Silicon wafers of size 5 × 5 mm^2^ (Agar Scientific Ltd) were cleaned by room temperature sonication in ethanol (≈ 10 mL, 30 min, Merck) and chloroform (≈ 10 mL, 60 min, Honeywell). After sonication, wafers were stored in pure chloroform solution. The wafers were allowed to air dry immediately prior to spin coating. A 100 mM solution of cholesterol (Sigma-Aldrich) was prepared in chloroform. A spin coater (Laurell Technologies, US) was used to prepare homogenous cholesterol films. Cholesterol deposition was done by drop-casting the standards on a silicon wafer in dynamic mode at the speed of 1800 rpm. A total of 70 μL was added to the wafer using a micropipette. The films were allowed to spin until the surface was a dry homogeneous film which could be visually observed. The films were then attached to a sample stub (Ionoptika Ltd, UK) by conductive carbon tape.

### Mouse and rat brain tissue preparation and analysis

Mouse and rat brains (wild-type mice and Wistar rats) were obtained from the Wolfson Molecular Imaging Centre (University of Manchester, UK). The organs were collected in accordance with ethical guidelines described by the Animals (Scientific Procedures) Act 1986 and the UK Home Office. Prior to sectioning, the brains were snap-frozen in isopentane cooled with liquid nitrogen at −60 °C and kept in the freezer at −80 °C. Sectioning was done using a micro cryostat (Leica CM3050 S). A 15% carboxymethyl cellulose (Sigma-Aldrich) matrix was used to attach the brain to the microtome stand. The sectioning was done at −20 °C. Brain is cut into sagittal sections of approximately 10–12 μm thickness. The sections were thaw-mounted onto Si wafers for mice and larger indium tin oxide (ITO) coated glass slides (Sigma-Aldrich) for rats and kept in the −80 °C freezer. The sample processing procedure is similar to that described in Fig. 1 of Yoon et al. [45]. Tissue samples were desiccated for 20 min prior to analysis. Sections were then attached to a sample stub and transferred into the ToF-SIMS instrument. Frozen-hydrated tissues which were mounted under liquid nitrogen directly from −80 °C storage and transferred to the ToF-SIMS maintaining cryogenic and low-humidity conditions. Following transfer to the instrument it was necessary to bring the temperature up to ≈ −60 °C under vacuum (between 10^−6^ and 10^−8^ mbar) in order to remove the ice that was formed on the surface of the tissue. The samples were maintained at ≈ −180 °C for the duration of the ToF-SIMS experiment. ToF-SIMS analysis was done in the same way as for the room temperature samples. Note that mouse brains were used for cholesterol imaging and quantification, while rat brains were used for depth profiling and cryogenic studies. This was done both due to tissue availability and due to the large size of rat brains which would have made imaging more demanding.

### ToF-SIMS analysis

Analysis was performed on a J105 ToF-SIMS (Ionoptika Ltd). 40-keV C_60_ and 70-keV (argon/carbon dioxide and water) gas cluster ion beams (GCIBs) were used for SIMS analysis. By adjusting the GCIB gun parameters it was possible to tune and mass select the clusters to a desired size [46]. The (Ar/CO_2_)_n_ mixture (Argoshield Universal, BOC) used for this study (both as the primary ion and as the carrier gas for water clusters) was composed of: Ar = 86%, CO_2_ = 12%, and O_2_ 2%. For the cholesterol standard a range of cluster sizes for both cluster species was tested (Table 1). The ion dose used for a single layer analysis was 5 × 10^11^ ions/cm^2^ in an area of 400 × 400 μm^2^. Cholesterol standard samples were analysed as depth profiles with 3 layers. Analysis parameters used for tissue imaging are shown in Table 2. Additionally, extended depth profiles were collected on rat brains with ion dose of approximately 1 × 10^13^ ions/cm^2^ per layer, with a total of 1000 layers. This was done in different brain regions with (ArCO_2_)_*n*_ clusters (cluster size (*n*) between 6400 and 6500).

**Table 1:** Average GCIB cluster sizes used for cholesterol standards analysis. Corresponding *E/m* values were calculated using the cluster size data. Showing information for both Ar/CO_2_ and H_2_O clusters

Ar/CO_2_			
Positive		Negative	
Average cluster size	E/m (eV/u)	Average cluster size	E/m (eV/u)
17,131	0.100	17,076	0.100
12,455	0.137	12,210	0.140
8579	0.199	8512	0.201
6886	0.248	6820	0.250
4819	0.354	4805	0.355
3403	0.502	3409	0.501
H_2_O			
Positive		Negative	
Average cluster size	E/m (eV/u)	Average cluster size	E/m (eV/u)
37,010	0.105	38,804	0.100
28,161	0.138	27,903	0.139
19,326	0.201	19,326	0.201
15,579	0.250	15,771	0.247
11,156	0.349	11,237	0.346
7873	0.494	7737	0.503

**Table 2:** Showing the parameters used for mass spectrometry imaging of mouse brains

	C_60_		ArCO_2_		H_2_O	
Ion mode	Positive	Negative	Positive	Negative	Positive	Negative
Cluster size	60	60	5900	5100	20,000	20,000
Spot size (µm)	5.7	5.7	6.6	8.8	8	8
Raster area (µm^2^)	450 × 450	450 × 450	450 × 450	450 × 450	450 × 450	500 × 500
Pixels in raster area	64 × 64	64 × 64	64 × 64	64 × 64	64 × 64	64 × 64
Ion dose (ions/cm^2^)	1.0E + 12	1.0E + 12	1.0E + 12	1.0E + 12	1.0E + 12	1.1E + 12

### **Data processing **

Mass spectrometry data was analysed using the Image Analyser software (Ionoptika Ltd, UK). Triplicate analyses were performed on the pure cholesterol films. Peaks of interest were selected for comparative quantification of primary ion efficiencies. For cholesterol, these peaks were *m/z* 369.353 ([M+H-H_2_O]^+^; −2.37 ppm difference from theoretical mass) and *m/z* 385.375 ([M-H]^-^; −72.56 ppm difference from theoretical mass), in positive and negative ion mode respectively. The peaks were selected with a range of 0.2 mass units (amu). Signal intensity (area under the curve) was summed for the three layers that were analysed. The procedure was repeated for all primary ion beams and all the cluster sizes. The signal intensity was divided by the primary ion dose to determine secondary ion yield (SIY).

The mass spectrometry images for cholesterol were obtained by selecting the corresponding peaks in both positive and negative ion mode. These images were then used to determine the intensity range, bin the image pixels based on their relative intensity, exclude outliers at the tails of the distribution that have only 1 pixel with the given intensity, and then linearly adjust the colour map. The linear adjustment was done in a way where all of the excluded pixel intensities got converted to the newly set maximum or minimum (depending on if they were excluded for high signal or low signal). In this way the image colour map was not overly skewed by the presence of a small number of very bright or dark pixels. The mass spectrometry images of the *cerebellum *were extracted from the whole brain images. These mass spectrometry images of the region of interest (ROI) were then processed using python (Spyder environment in Anaconda). The pixels that had intensity in the top and bottom 2% were clipped. This was done so to remove pixel intensity outliers. The image was then normalised and intensities between 0 and 1 were obtained for each pixel. The image was then replotted using the normalised intensities. The spatial resolution of the clusters was measured by using intensity line scans on these images. This was done using FIJI (ImageJ) [47]. The 20%–80% intensity criterion was applied to spatial resolution measurements. Ten line scans were made for each of 6 samples (three PIs in both polarities).

To obtain further information on the cholesterol yields and matrix effects in the brain, two regions of interest from the whole brain images were selected, the *corpus callosum* (CC) and *cerebral cortex*. Five separate areas were selected in both of these brain regions using an 11 × 11-pixel selection tool. The area under the curve was calculated and the resulting values were used to compare the yields and matrix effects of the three PIs used to collect the images. Data obtained from samples analysed under cryogenic conditions was processed in a similar way except areas of two separate brain sections were each analysed with both (ArCO_2_)_n_ and (H_2_O)_n_ clusters. In each sample, separate but adjacent areas of the brain were imaged with each PI. The whole mass spectrometry image was used to derive the signal intensities. These intensities were normalised to the total ion (TI) intensity. Experimental information for cryogenic imaging experiments is shown in Table S1.

## Results and discussion

### Mechanism of ion formation

**Fig. 1:**
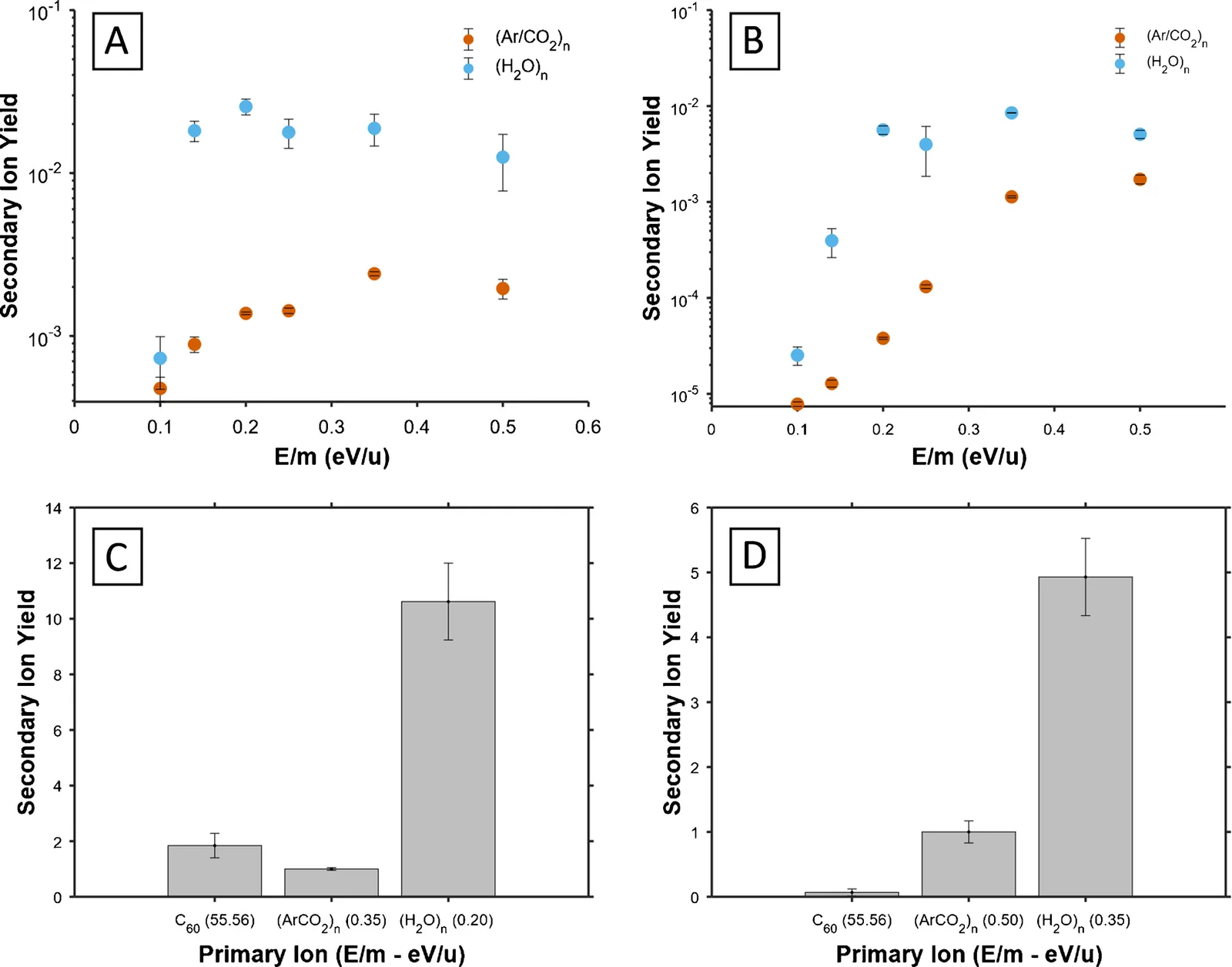
**A** and **B** showing the dependence of cholesterol molecular ion Secondary Ion Yield on the *E/m* of primary ion cluster used for the analysis in positive and negative ion mode respectively. **C** and **D** compare the highest measured Relative SIY for the given primary ion in positive and negative ion mode respectively. Peak at *m/z* 369.353 [M+H-H_2_O]^+^ was analysed in positive ion mode (**A** and **C**) while *m/z* 385.375 [M-H]^-^ was analysed in negative ion mode (**B** and **D**)

It has previously been shown that the *E/m *parameter (energy per mass unit, a measure of velocity) has a significant effect on the yields resulting from primary ion impact [26, 40, 48, 49]. The chemical and physical heterogeneity of tissue samples results in significant challenges for quantitative method development. Therefore, a pure cholesterol standard was analysed in order to determine which primary ion beams would be optimal for cholesterol analysis. We acknowledge that the physical as well as the chemical nature of the sample will have an effect on the relative ion yield of different primary ion beams and yields from thick films will not necessarily reflect those from tissue matrices. However, the cholesterol standard film represents a more reproducible reference material than a heterogeneous tissue. Based on previous research [26, 40, 49], a range of different *E/m* values were chosen (Table 1) and corresponding cluster sizes tested with both (Ar/CO_2_)_n_ and (H_2_O)_n_ PIs. These were also compared to our benchmark of C_60_ yields. From this data it could be observed that in both positive and negative ion mode it was the (H_2_O)_n_ clusters that resulted in higher secondary ion yield for all *E/m* values tested, when compared to (Ar/CO_2_)_n_ (Fig. 1). This is in agreement with previous studies on similar sized analytes [26, 40, 43, 50].

The highest SIY was observed at *E/m* values of 0.20 and 0.35 eV/u for (H_2_O)_n_ in positive and negative ion mode respectively (Fig. 1A and B). For (Ar/CO_2_)_n_, corresponding maxima were recorded at *E/m* of 0.35 and 0.5 eV/u in positive and negative ion mode respectively (Fig. 1A and B). This discrepancy between optimal *E/m *for different primary ion clusters has been recorded previously [40, 51]. Furthermore, for (H_2_O)_n_ clusters, a decrease in yield was observed at both extremes (0.1 and 0.5 eV/u). On the other hand, it was observed that for (Ar/CO_2_)_n_ clusters the maximum had shifted to comparatively higher *E/m* values. This implied a further potential increase in yield with greater *E/m* values for (ArCO_2_)_n_ clusters. This information indicated that for these standard samples, water and argon/carbon dioxide clusters of the same energy per mass unit (velocity) showed differing behaviours with regards to yield. This is likely to be caused by a mixture of sputter and ionisation yield differences for these clusters.

For both cluster species, the reduced SIYs at the lowest *E/m *values explored in this study (< 0.2 eV/u) can be explained by the lack of required energy to break the analyte intermolecular forces and cause sputtering of the secondary ions, resulting in reduced sputter yields at lower cluster velocity [52–54]. Additionally, the physical and chemical nature of the sample and the surface play important roles in the sputtering process [55–57]. However, the differences between SIYs of different GCIBs became more pronounced just above the sputtering threshold at *E/m* values ~ 0.2–0.5 eV/u, which presents an opportunity for further experimentation and investigation into potentially differing GCIB ionisation mechanisms. Previous results [40, 51] have shown that for other compounds a different pattern is observed, where at higher *E/m* values there is convergence of SIYs between water and argon clusters. This points to the fact that the overall yield and the optimal *E/m* range will greatly depend on the nature of the analytes. Another potential explanation for the difference in optimal *E/m* is that for (Ar/CO_2_)_n_ and (H_2_O)_n_ clusters the energies per atom/molecule (*E/n*) were quite different which could affect the bond dissociation probabilities for these molecules. The maximum *E/n* value tested for (Ar/CO_2_)_n_ clusters was 20.6 eV/molecule while for (H_2_O)_n_ it was only 9 eV/molecule (these clusters having approximately matched velocity — *E/m*). Additionally, there is evidence that when analysing thin organic films using argon clusters, the value of 5 eV/atom acts as a turning point above which much greater degree of analyte fragmentation occurs [58]. While these values are analyte-dependent [59], they still present a potential difference in yield caused by a change in bond breaking within the clusters as well as the fragmentation rate of the analyte. This can be observed for water cluster fragmentation ratios (Figure S7A). In positive ion mode the plateau for degree of fragmentation starts at approximately 0.3 eV/u. When converted to the same units, the above mentioned value of 5 eV/molecule is approximately 0.28 eV/u, which corresponds quite well to the empirical data. However, in negative ion mode this phenomenon is not observed and there might be other explanations for the fragmentation ratio (different ionisation and fragmentation mechanisms). Additionally, experiments were done that show that even at relatively high *E/m* values (0.35 eV/u) for both cluster types, the fragmentation is significantly lower than that for C_60_ (Figure S8).

One potential explanation for why the water clusters had higher yields is that upon impact, a plume of ionised water molecules and/or fragments is created close to the sample surface. This is akin to the plume created during laser desorption with MALDI. An increase in density of the plume increases the chances of ion-molecule collision and thus results in greater ionisation probabilities [60, 61]. Similarly with water clusters, the longer the sputtered molecules spend in the plume the greater the chance of ion-molecule reactions, e.g. proton attachment/transfer from the water GCIB constituents to the analyte. It had previously been described that PIs with higher *E/m* values exhibit a notably higher velocity of pressure wave propagation upon impact compared to those with lower *E/m *values [59]. This implies that the PIs with lower *E/m* result in slower cluster impacts and thus potentially greater plume dwell time of PI fragments that might contribute to SI formation. This would result in lowered ionisation efficiency at high *E/m *values when using GCIBs with reactive components such as water. This was further supported by molecular dynamics (MD) simulations which show that at both very low and very high cluster sizes the trehalose yields are reduced [61]. For low *E/m* values (large clusters) the trehalose does not sputter but rather the water clusters undergo a soft landing, dissolving the trehalose in the process and forming a nanodroplet. At high *E/m* value the water molecules backscatter too quickly and have lower chances of forming a trehalose-water complex.

It is for this reason that the values in the middle range of *E/m *are optimal; these clusters both have enough energy for sputtering and are slow enough to allow for water to interact with the sputtered analyte, thus increasing the chances of proton transfer [61]. (Ar/CO_2_)_n_ clusters may depend on an alternative mechanism for secondary ion formation, such as direct impact-induced processes, which could also serve to explain the greater yields for (ArCO_2_)_n_ at higher *E/m* values compared to water clusters. Promotion of oxygen adduct ions has also been observed in the analysis of organic and inorganic samples using CO_2 _GCIBs [62], which might be a compounding variable that complicates this analysis. The data presented here also show that the SIY for C_60_ primary ions was lower compared to water clusters (Fig. 1C and D). The difference between water and C_60_ yields could result from differences in sputter yield and/or ionisation efficiency, as well as the degree of analyte fragmentation (Figures S7 and S8). Compared to the GCIB primary ions used in this research, the C_60_ has a much higher *E/m* value (≈55.56 eV/u). This more energetic impact on the surface results in a greater degree of bond fragmentation and more surface damage. In positive ion mode, the cholesterol [M+H-H_2_O]^+^ yield from C_60_ was higher than from (ArCO_2_)_n_ (Fig. 1C)_,_ while the opposite was true in negative ion mode for [M-H]^-^ (Fig. 1D). This may be related to the energetics of the formation of the secondary ions or reflect the different chemistry of the primary ions. The cluster PI composition was shown to be greatly dependent on the input gases. Experiments have shown that there was not a linear correlation between the ratio of input gases and the resulting composition of the clusters [44, 63]. Due to this, the (ArCO_2_)_n_ clusters that were used in this experiment were likely to be majority CO_2_ with minor contributions from Ar. However, since the exact PI composition was not precisely determined for this experiment, there is some potential uncertainty over the exact cluster composition.

It was observed that the ion yield ratio of positive and negative pseudo-molecular cholesterol species for C_60_, (ArCO_2_)_n_, and (H_2_O)_n_ was 38.32 (± 27.74), 1.40 (± 0.15), and 3.01 (± 0.34) respectively. For each cluster the *E/m* values that were compared were those where the maximum yield was observed. For (ArCO_2_)_n_ clusters this was at 0.35 eV/u (positive) and 0.5 eV/u (negative), while for the (H_2_O)_n_ clusters it was 0.2 eV/u (positive) and 0.35 eV/u (negative). With regard to observed ratios for (ArCO_2_)_n_ and (H_2_O)_n_, there is likely a cluster chemistry-dependent effect. For water clusters this was potentially acid/base related which would imply dependence on the gas-phase basicity of the analyte [42]. This was especially likely due to the potential acidic nature of the water clusters due to the mixing with the carbon dioxide in the carrier gas. This would result in a cluster that was a better proton donor, thus increasing the chances of protonation and resulting in higher positive to negative ion yield ratios. These types of cluster-based differences in positive and negative SYI were previously observed when using CO_2_-doped water clusters [43].

### Tissue analysis

**Fig. 2:**
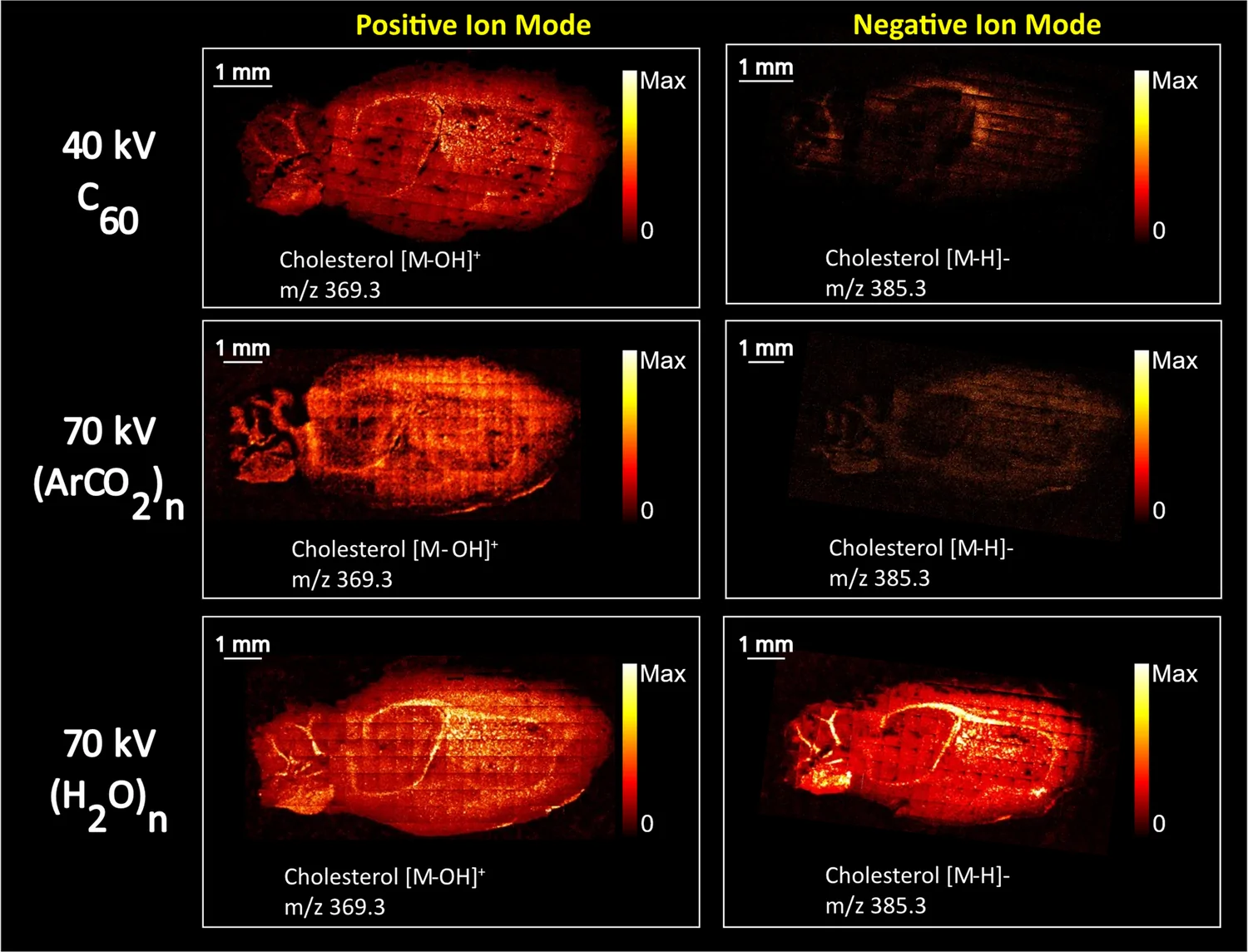
ToF-SIMS images of freeze-dried mouse brain sections analysed at room temperature with different primary ion beam identities and cluster sizes. Data for both positive and negative ion mode are shown. This comparison shows the extent of the improvement in yield that results from using water clusters to analyse biological samples. Note that the images have been linearly scaled to exclude outliers with signal intensities that were either too high or too low. Scale bars were estimated from the known size of analysis areas. For more information about this procedure see the methods section

**Fig. 3:**
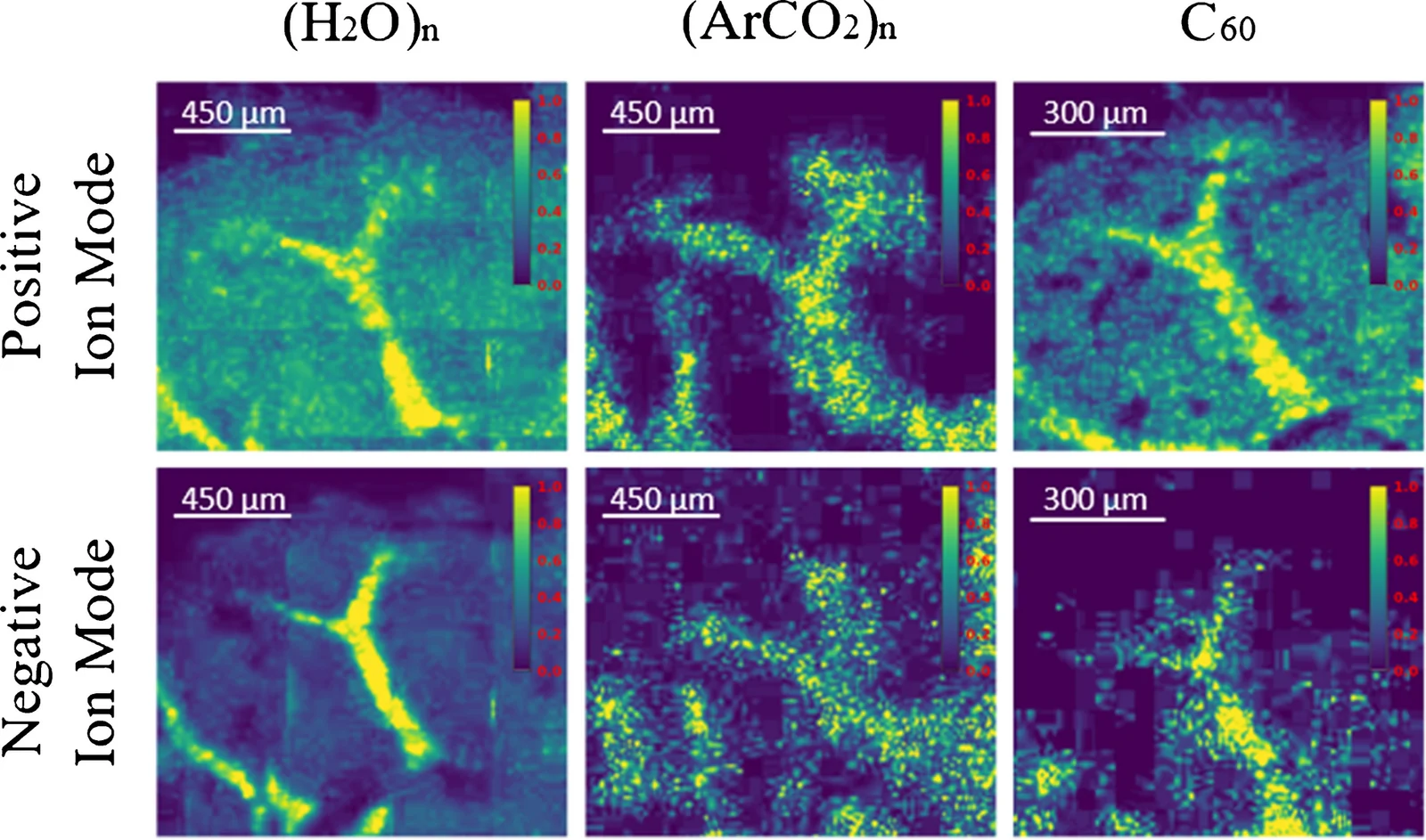
ToF-SIMS images of cholesterol in the cerebellum of freeze-dried mouse brain sections analysed at room temperature. Positive ion mode — *m/z* 369.3; Negative ion mode — *m/z* 385.3. Note that the images have been linearly scaled to exclude outliers with signal intensities that were either too high or too low. For more information about this procedure see the methods section. Additionally, these images were obtained from the whole brain cholesterol ion images shown in Fig. 2. The images were normalised in accordance with observed pixel brightness. Scale bars were estimated from the known size of analysis areas

**Fig. 4:**
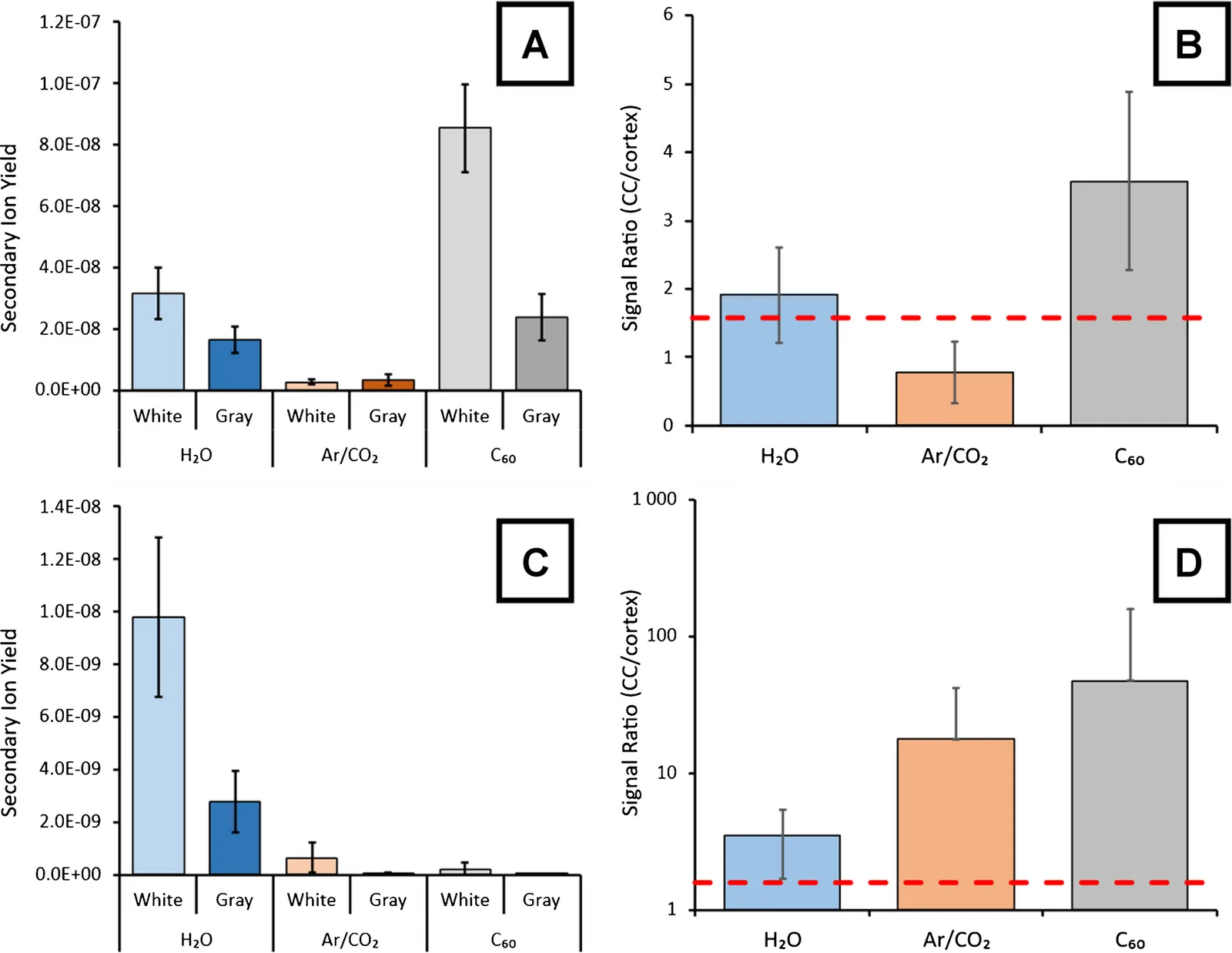
Cholesterol secondary ion yield of three different primary ion sources in white (corpus callosum) and grey (cortex) matter of mouse brain in positive (**A**, **B**) and negative (**C**, **D**) ion mode. Ions measured in panels **A** and **C** are *m/z* 369 [M+H-H_2_O]^+^ and *m/z* 385 [M-H]^-^ respectively. **B** and **D** show the ratio of cholesterol signal intensity in CC and cortex. Note that the dashed red line represents a literature value for the ratio of cholesterol concentrations (CC/cortex) [18]

Whole mouse brain sections were imaged with the goal of exploring the distribution of cholesterol in the tissue and assessing the efficiency of the PIs for biological sample analysis. Three different primary ions were applied with almost identical primary ion dose for analysis of cholesterol in mouse brain: C_60_, (ArCO_2_)_n_, and (H_2_O)_n_ (Figs. 2, 3, 4, and S1). Based on pure cholesterol data it was established which *E/m* values result in the highest yields from GCIB projectiles (*E/m* = 0.20 eV/u for (H_2_O)_n_ clusters and *E/m* = 0.35 eV/u for (Ar/CO_2_)_n_ clusters). The signal intensity and image quality were significantly different for the tested primary ions. At optimum *E/m* values, mass spectra (Figure S1) showed clear differences between the various PIs in both polarities, with (H_2_O)_n_ clusters producing a marked shift to higher *m/z *values for detected ions. This was likely due to the softer nature of ion impact as well as higher chances of lipid ionisation with water clusters [26]. The zoomed in spectra (Figures S2 and S3) showed the enhancement in lipid SIY using (H_2_O)_n_ clusters in both positive and negative ion mode within the ‘lipid region’ *m/z* 700–900.

In positive ion mode, the cholesterol ion images obtained using C_60_ and (H_2_O)_n_ primary ions appeared similar (Figs. 2 and 3). However, the cholesterol signal in white matter was more consistent when imaged with water clusters, particularly in regions such as the *corpus callosum* (CC) and *cerebellum*. Despite C_60_ having a smaller pixel size in this experiment, the cholesterol images produced by water clusters exhibited comparable contrast and sharpness (Figure S9). In contrast, (ArCO_2_)_n_ clusters yielded both lower signal intensity (Fig. 4C) and poorer spatial resolution (Figs. 2 and 3). These qualitative observations were further supported by spatial resolution measurements using signal intensity line scans across white matter features in the *cerebellum*. Applying the 20%–80% intensity criterion, the results (Figure S9) show that in positive ion mode (H_2_O)_n_ provides slightly better spatial resolution than C_60_, while both outperform (ArCO_2_)_n_ clusters. In negative ion mode, (H_2_O)_n_ clusters demonstrated a clear improvement in spatial resolution compared to both C_60_ and (ArCO_2_)_n_, which exhibited nearly identical performance. Additionally, spot size measurements for C_60_ and (ArCO_2_)_n_ showed larger error bars, suggesting greater variability in the mass spectrometry images acquired with these primary ions (Fig. 3).

Of particular interest is the distribution of cholesterol signal between anatomical regions and how this compares with quantitative data. The grey matter in the *cerebellum* was very low in cholesterol signal when imaged with (ArCO_2_)_n_. On the other hand, when imaged with both other PIs, cholesterol signal was observed throughout the whole *cerebellum*, as well as the whole brain. This is further explored by sampling areas in the cortex (grey matter) and the adjacent *corpus callosum* (white matter) separately to determine relative cholesterol yields from each anatomical region. This analysis was done by selecting five separate areas in each of the regions of interest and averaging the signal over a number of pixels (11 × 11) in those areas. In positive ion mode, the signal intensity for C_60_ was the highest (Fig. 4A), which could likely be explained by a significantly higher *E/m* value and the associated higher sputter yield. (ArCO_2_)_n_ showed significantly lower yields in both of the analysed brain areas. The SIY data shown in Fig. 4C supported the descriptive observations from Figs. 2 and 3. In negative ion mode, water clusters significantly increased the detected cholesterol intensity in both the grey and the white brain matter, as compared to the signal intensity for (ArCO_2_)_n_ and C_60_. With these two PIs, there were low signals of cholesterol detected in the grey matter, which was in line with previous research that implied cholesterol localised solely in white brain matter tissue (Bi_3_primary beam in negative ion mode) [23]. Water cluster data presented here showed that cholesterol could be clearly detected in both white and grey matter, as well as covering the whole area of the brain section with varying degrees of intensity (Fig. 2).

Additionally, ratios of cholesterol signal intensities in the CC (white brain matter) and cortex (grey brain matter) were compared for the three PIs (Fig. 4B—positive ion mode, Fig. 4D—negative ion mode). The red line in these figures represents the ratio of cholesterol concentrations (CC/cortex) reported in a previous publication [18]. In that study, Angelini et al. used an enzyme-assisted derivatisation for sterol analysis (EADSA) methodology to derivatise cholesterol in brain sections, allowing for a quantitative analysis using MALDI mass spectrometry. Our dataset using (H_2_O)_n_PIs showed that in both positive and negative ion modes the ratio of concentrations was closest to the literature value when compared to the two other tested PIs. This ability of water clusters to reduce matrix effects had already been reported in biomolecular mixtures [31, 44, 64]. It is worth noting that in the studies by Angelini et al. and Nezhad et al., which focused on cholesterol quantification, [18, 65] extensive sample preparation was required (derivatisation and silver coating, respectively) to enhance cholesterol ionisation. In contrast, the use of water clusters in the study presented here required no sample preparation, thereby minimising the risk of additional matrix effects or analyte redistribution, and making experiments more time efficient.

In this study it is shown that even in very complex biological samples water clusters resulted in cholesterol distribution data that more closely resembles quantitative literature data [18]. Noting the logarithmic scale, the large error bars for (ArCO_2_)_n_ and C_60_ analyses in Fig. 4D were due to very low signal intensities measured using these PIs in negative ion mode (Fig. 4C). This implied that in negative ion mode analysis of brain tissue both of these PIs were not very useful and should be avoided, especially for analysis of cholesterol and/or related lipid molecules.

**Fig. 5:**
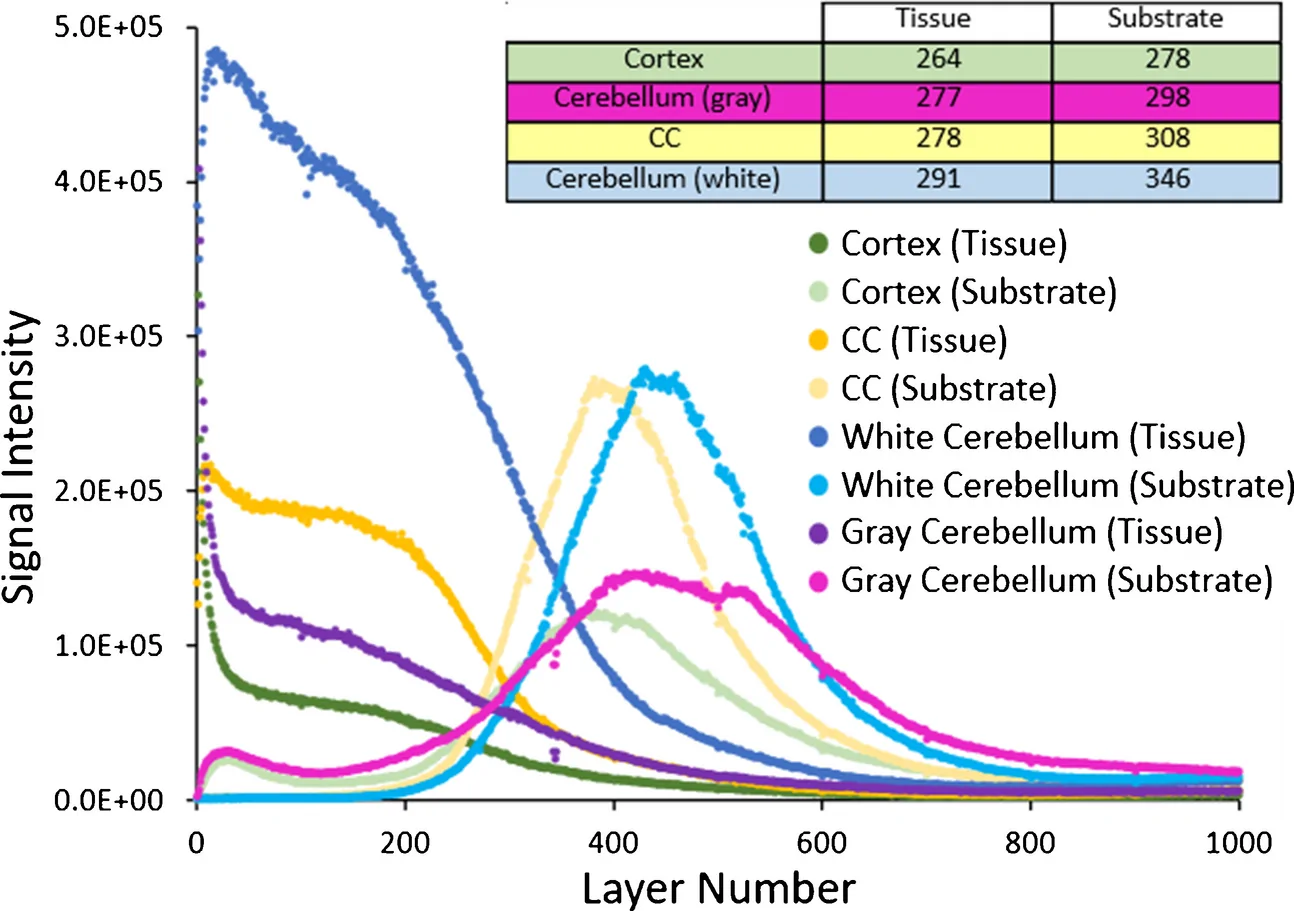
Depth profiles for different rat brain regions collected using ArCO_2_ clusters. The tissue peak measured is *m/z* 86.1 and the substrate is *m/z* 114.9. The substrate peak comes from indium ions that are sputtered from the ITO slide indicating that the brain has been sputtered completely. The inserted table shows the layers at which both the tissue and substrate signals reach 50% of their maximum value. Note that for 100% signal intensity, maximum detected value was used for substrate and value at layer 50 was used for the tissue

The recorded increase in yields could be caused by an increase in sputter yields and/or ionisation efficiency, which may differ between regions in a heterogeneous tissue such as the brain. It is important to try to untangle these two phenomena in seeking to better understand the performance characteristics of different ion beams and samples. Based on previous research (universal sputter rate curve for Ar GCIBs) it was found that one of the most important factors determining the sputter rate in cluster primary ions was the *E/m *value [48, 49]. For our analyses we used (H_2_O)_n_ and (Ar/CO_2_)_n_ clusters with closely matched *E/m*. By analysing depth profile data, conclusions could be drawn about the sputter rates in various brain regions (Fig. 5). An abundant organic ion at *m/z* 86.1 (choline headgroup fragment) was taken as an indication of tissue sputtering. The indium peak at *m/z* 114.9 was used as a representation of the ITO slide substrate. The same (Ar/CO_2_)_n_ cluster size and ion dose was used to collect the depth profiles in all the regions (Fig. 5). Based on this data it could be observed that different brain regions had differing sputter rates. This is indicated by the 50% signal points (inlay table in Fig. 5). Both the 50% signal levels of tissue and substrate provide a consistent indication that sputter rate (*cortex* > *cerebellum* (grey) ≈ *corpus callosum* > *cerebellum* (white)).

In general, both grey brain matter regions (*Cerebellum* (grey) and *cortex*) had higher sputter rates and reached the substrate at a faster rate compared to the white brain matter regions (*Cerebellum* (white) and CC). This is something that should be taken into consideration when attempting to extract quantitative information from ToF-SIMS analysis of brains and by implication other heterogeneous tissues. Further AFM studies could provide a more accurate indication of the extent of difference in sputter rates; however, our attempts to achieve this were unsuccessful due to the varied topography of the tissue.

**Fig. 6:**
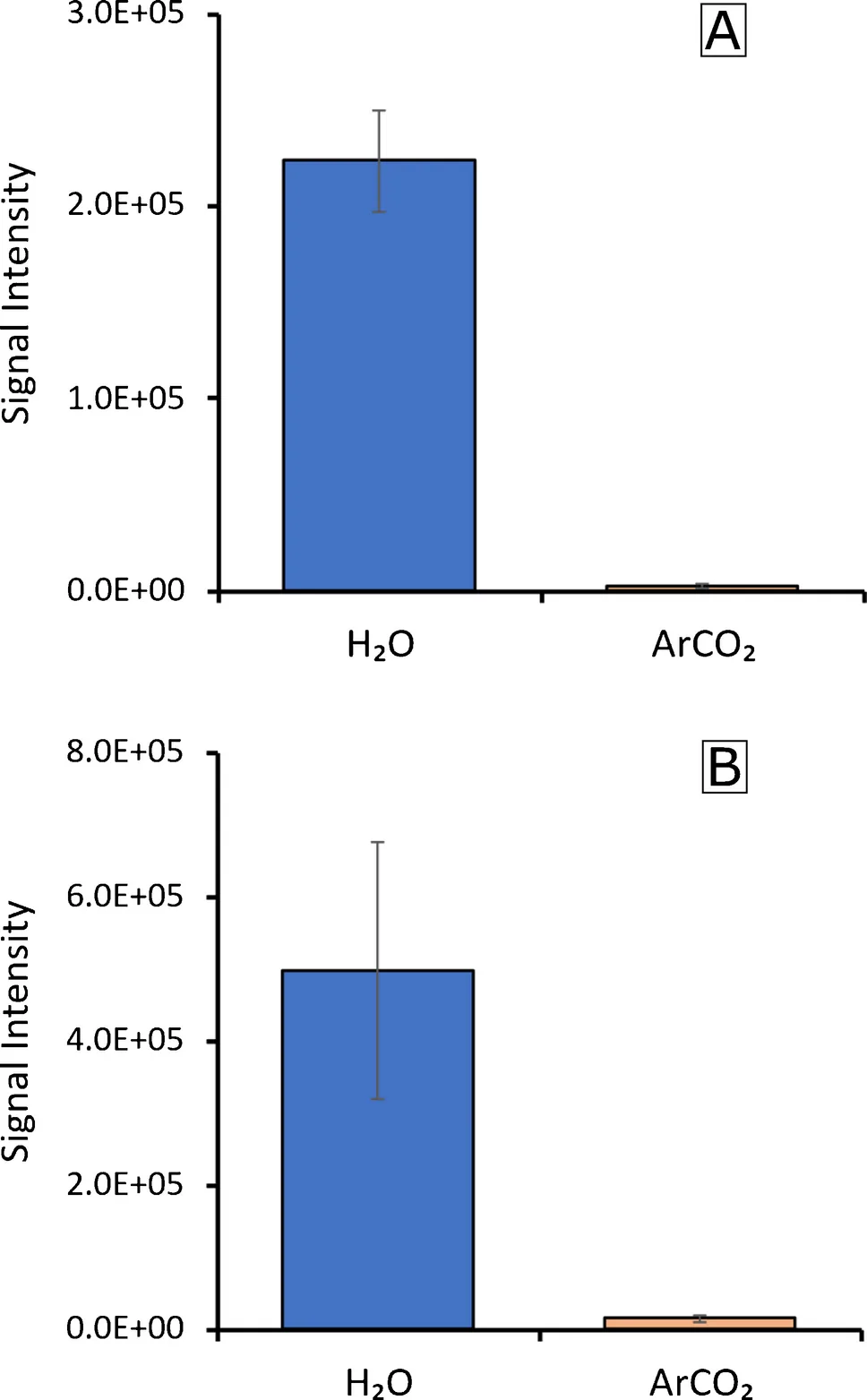
Comparison of cholesterol signal intensities in rat brain sections under cryogenic conditions using different GCIBs. **A** — *m/z* 369 [M+H-H_2_O]^+^; **B** — *m/z* 385 [M-H]^-^

There are a range of benefits in applying cryogenic conditions to ToF-SIMS analysis, including reduction or prevention of molecular migration [6, 38], reduced damage accumulation [66] and increase in yield [66–69]. Due to these advantages cryogenic conditions have been applied to a variety of ToF-SIMS analyses including cells in 3D [19], breast cancer tissue [70], bacterial biofilms [71], cholesterol in macrophage cells [72], etc. In this study rat brain sections were analysed under cryogenic conditions. This was done with the goal of comparing the ToF-SIMS performance of (H_2_O)_n_ and (ArCO_2_)_n_ clusters. Under frozen-hydrated conditions it was hypothesised that (ArCO_2_)_n_ clusters would benefit to a greater degree from ionisation enhancement effect, due to increased concentration of hydrogen ions in the sputter crater/plume from fragmented ice matrix molecules. However, it was clear that the water clusters still resulted in significantly greater cholesterol secondary ion yields (Fig. 6). This supported the findings of Sheraz et al., which showed that under these beam parameters cryofixation does not increase yields when paired with a (CO_2_)_n _beam [50]. It should be noted that sublimation (at −60 °C in the preparation chamber) was employed to remove surface ice which would mask surface chemistry. Detection of the surface ice present during cryogenic analysis (Figure S4) showed that there was significantly higher water signal with (H_2_O)_n_ *vs*. (ArCO_2_)_n_ clusters. While there are slight differences between positive and negative ion mode in both cases the (ArCO_2_)_n_ clusters show significantly lower water trimer signal.

Lastly, if normalised to total ion intensity the signal intensities for cholesterol in cryogenic tissue are still higher using (H_2_O)_n_ while other lipids showed significantly different behaviour (Figure S4). Note that the raw signal (not total ion normalised) outputs for water clusters were still significantly higher. These findings imply that the ionisation efficiency of cholesterol differs significantly from that of many other lipid molecules, suggesting that (H_2_O)_n_ may be particularly well suited for cholesterol analysis. Cryogenic analysis of tissue has additional experimental challenges associated with it. Sample handling is significantly more complex, experiments may be time sensitive due to limited supply of liquid nitrogen, and ice-related charging effects may require different charge compensation strategies during the course of an analysis.

## Conclusion

The useful yield of cholesterol analysis in both pure standards and in murine brain is shown to be greatly dependent on the primary ion cluster chemistry as well as the energy per mass unit (velocity) of the cluster. It is found that water clusters result in greater cholesterol yields than either the (Ar/CO_2_)_n_ clusters or C_60_ primary ions. This yield increase for water clusters is significant in both positive and negative ion mode in pure cholesterol, while in murine brain tissue the benefits due to use of water clusters are most prominent in negative ion mode. The application of water clusters to biological tissue has been shown to result in increased signal intensity for cholesterol (including for frozen-hydrated samples), higher-resolution, and more quantitative molecular imaging capabilities. This presents a contribution to MSI techniques since in the past, imaging of cholesterol has presented a major obstacle for other mass spectrometry techniques. Collectively, these advances provide opportunities for more reliable and quantitative in situ molecular measurements of heterogeneous tissue samples using ToF-SIMS.

## Supplementary Information

Below is the link to the electronic supplementary material.

ESM 1 Supplementary Material 1 (DOCX 4.74 MB)

## Data Availability

The data that support the findings of this study are available from the corresponding author upon reasonable request.

## Declarations

Mouse and rat brains (wild-type mice and Wistar rats) were obtained from the Wolfson Molecular Imaging Centre (University of Manchester, UK). The organs were collected in accordance with ethical guidelines described by the Animals (Scientific Procedures) Act 1986 and the UK Home Office.

## References

[1] Sonal Sekhar M, Marupuru S, Reddy BS, Kurian SJ, Rao M. Physiological Role of Cholesterol in Human Body. In: Dietary Sugar, Salt and Fat in Human Health. Elsevier; 2020. p. 453–81. 10.1016/B978-0-12-816918-6.00021-4.

[2] Dietschy JM, Turley SD. Cholesterol metabolism in the central nervous system during early development and in the mature animal. J Lipid Res. 2004;45(8):1375–97. 10.1194/jlr.R400004-JLR200. 15254070

[3] Quan G, Xie C, Dietschy JM, Turley SD. Ontogenesis and regulation of cholesterol metabolism in the central nervous system of the mouse. Dev Brain Res. 2003;146(1–2):87–98. 10.1016/j.devbrainres.2003.09.015. 14643015

[4] Saher G, Brügger B, Lappe-Siefke C, Möbius W, Tozawa R, Wehr MC, et al. High cholesterol level is essential for myelin membrane growth. Nat Neurosci. 2005;8(4):468–75. 10.1038/nn1426. 15793579

[5] Saher G, Quintes S, Nave K-A. Cholesterol: a novel regulatory role in myelin formation. Neuroscientist. 2011;17(1):79–93. 10.1177/1073858410373835. 21343408

[6] Sjövall P, Johansson B, Lausmaa J. Localization of lipids in freeze-dried mouse brain sections by imaging TOF-SIMS. Appl Surf Sci. 2006;252(19):6966–74. 10.1016/j.apsusc.2006.02.126.

[7] Korade Z, Kenworthy AK. Lipid rafts, cholesterol, and the brain. Neuropharmacology. 2008;55(8):1265–73. 10.1016/j.neuropharm.2008.02.019. PMC2638588 18402986

[8] Ohvo-Rekilä H, Ramstedt B, Leppimäki P, Slotte JP. Cholesterol interactions with phospholipids in membranes. Prog Lipid Res. 2002;41(1):66–97. 10.1016/S0163-7827(01)00020-0. 11694269

[9] Incardona JP, Eaton S. Cholesterol in signal transduction. Curr Opin Cell Biol. 2000;12(2):193–203. 10.1016/S0955-0674(99)00076-9. 10712926

[10] Jin U, Park SJ, Park SM. Cholesterol metabolism in the brain and its association with Parkinson’s disease. Exp Neurobiol. 2019;28(5):554–67. 10.5607/en.2019.28.5.554. PMC6844833 31698548

[11] Cha S-H, Choi YR, Heo C-H, Kang S-J, Joe E-H, Jou I, et al. Loss of parkin promotes lipid rafts-dependent endocytosis through accumulating caveolin-1: implications for Parkinson’s disease. Mol Neurodegener. 2015;10(1):63. 10.1186/s13024-015-0060-5. PMC4666086 26627850

[12] Cutler RG, Kelly J, Storie K, Pedersen WA, Tammara A, Hatanpaa K, et al. <article-title update="added">Involvement of oxidative stress-induced abnormalities in ceramide and cholesterol metabolism in brain aging and Alzheimer’s disease. Proc Natl Acad Sci. 2004;101(7):2070–5. 10.1073/pnas.0305799101. PMC357053 14970312

[13] Heverin M, Bogdanovic N, Lütjohann D, Bayer T, Pikuleva I, Bretillon L, et al. <article-title update="added">Changes in the levels of cerebral and extracerebral sterols in the brain of patients with Alzheimer’s disease. J Lipid Res. 2004;45(1):186–93. 10.1194/jlr.M300320-JLR200. 14523054

[14] Jones L, Holmans PA, Hamshere ML, Harold D, Moskvina V, Ivanov D, et al. <article-title update="added">Genetic evidence implicates the immune system and cholesterol metabolism in the aetiology of Alzheimer’s disease. PLoS One. 2010;5(11):e13950. 10.1371/journal.pone.0013950. PMC2981526 21085570

[15] Li T, Yin Y, Zhou Z, Qiu J, Liu W, Zhang X, et al. Ion mobility-based sterolomics reveals spatially and temporally distinctive sterol lipids in the mouse brain. Nat Commun. 2021;12(1):4343. 10.1038/s41467-021-24672-x. PMC8282640 34267224

[16] Sandhoff R, Brügger B, Jeckel D, Lehmann WD, Wieland FT. Determination of cholesterol at the low picomole level by nano-electrospray ionization tandem mass spectrometry. J Lipid Res. 1999;40(1):126–32. 9869658

[17] Gallego SF, Højlund K, Ejsing CS. Easy, fast, and reproducible quantification of cholesterol and other lipids in human plasma by combined high resolution MSX and FTMS analysis. J Am Soc Mass Spectrom. 2018;29(1):34–41. 10.1007/s13361-017-1829-2. 29063477

[18] Angelini R, Yutuc E, Wyatt MF, Newton J, Yusuf FA, Griffiths L, et al. Visualizing cholesterol in the brain by on-tissue derivatization and quantitative mass spectrometry imaging. Anal Chem. 2021;93(11):4932–43. 10.1021/acs.analchem.0c05399. PMC7992047 33687199

[19] Passarelli MK, Winograd N. Lipid imaging with time-of-flight secondary ion mass spectrometry (ToF-SIMS). Biochimica et Biophysica Acta (BBA) - Molecular and Cell Biology of Lipids. 2011;1811(11):976–90. 10.1016/j.bbalip.2011.05.007. PMC3199347 21664291

[20] Fletcher JS. Latest applications of 3D ToF-SIMS bio-imaging. Biointerphases. 2015;10(1):018902. 10.1116/1.4907727. 25708631

[21] Kompauer M, Heiles S, Spengler B. Atmospheric pressure MALDI mass spectrometry imaging of tissues and cells at 1.4-μm lateral resolution. Nat Methods. 2017;14(1):90–6. 10.1038/nmeth.4071. 27842060

[22] Nguyen SN, Sontag RL, Carson JP, Corley RA, Ansong C, Laskin J. Towards high-resolution tissue imaging using nanospray desorption electrospray ionization mass spectrometry coupled to shear force microscopy. J Am Soc Mass Spectrom. 2018;29(2):316–22. 10.1007/s13361-017-1750-8. PMC5787403 28755258

[23] Benabdellah F, Seyer A, Quinton L, Touboul D, Brunelle A, Laprévote O. Mass spectrometry imaging of rat brain sections: nanomolar sensitivity with MALDI versus nanometer resolution by TOF–SIMS. Anal Bioanal Chem. 2010;396(1):151–62. 10.1007/s00216-009-3031-2. 19711060

[24] Griffiths WJ, Yutuc E, Wang Y. Mass spectrometry imaging of cholesterol and oxysterols. In: Implication of Oxysterols and Phytosterols in Aging and Human Diseases; Lizard, G., Ed.; Advances in Experimental Medicine and Biology; Springer International Publishing: Cham, 2024;1440:73–87. 10.1007/978-3-031-43883-7_5. 38036876

[25] Berrueta Razo I, Sheraz S, Henderson A, Lockyer NP, Vickerman JC. Comparing C 60 + and (H 2 O) n + clusters for mouse brain tissue analysis. Surf Interface Anal. 2014;46(S1):136–9. 10.1002/sia.5597.

[26] Dimovska Nilsson K, Karagianni A, Kaya I, Henricsson M, Fletcher JS. (CO2)N+, (H2O)N+, and (H2O)N+ (CO2) gas cluster ion beam secondary ion mass spectrometry: analysis of lipid extracts, cells, and Alzheimer’s model mouse brain tissue. Anal Bioanal Chem. 2021;413(16):4181–94. 10.1007/s00216-021-03372-x. PMC8222020 33974088

[27] Appelhans AD, Delmore JE, Dahl DA. Focused, rasterable, high-energy neutral molecular beam probe for secondary ion mass spectrometry. Anal Chem. 1987;59(13):1685–91. 10.1021/ac00140a022.

[28] Weibel D, Wong S, Lockyer N, Blenkinsopp P, Hill R, Vickerman JC. A C 60 primary ion beam system for time of flight secondary ion mass spectrometry: its development and secondary ion yield characteristics. Anal Chem. 2003;75(7):1754–64. 10.1021/ac026338o. 12705613

[29] Yamada I, Matsuo J, Toyoda N, Kirkpatrick A. Materials processing by gas cluster ion beams. Mater Sci Eng R Rep. 2001;34(6):231–95. 10.1016/S0927-796X(01)00034-1.

[30] Yamada I, Takaoka GH, Current MI, Yamashita Y, Ishii M. Irradiation effects of gas-cluster CO2 ion beams on Si. Nucl Instrum Methods Phys Res Sect B Beam Interact Mater Atoms. 1993;74(1–2):341–6. 10.1016/0168-583X(93)95073-E.

[31] Sheraz née Rabbani S, Barber A, Fletcher JS, Lockyer NP, Vickerman JC. Enhancing secondary ion yields in time of flight-secondary ion mass spectrometry using water cluster primary beams. Anal Chem. 2013;85(12):5654–8. 10.1021/ac4013732. PMC3686110 23718847

[32] Piehowski PD, Carado AJ, Kurczy ME, Ostrowski SG, Heien ML, Winograd N, et al. MS/MS methodology to improve subcellular mapping of cholesterol using TOF-SIMS. Anal Chem. 2008;80(22):8662–7. 10.1021/ac801591r. PMC2597061 18925746

[33] Nygren H, Eriksson C, Malmberg P, Sahlin H, Carlsson L, Lausmaa J, et al. A cell preparation method allowing subcellular localization of cholesterol and phosphocholine with imaging TOF-SIMS. Colloids Surf B Biointerfaces. 2003;30(1–2):87–92. 10.1016/S0927-7765(03)00066-3.

[34] Nygren H, Malmberg P. Silver deposition on freeze-dried cells allows subcellular localization of cholesterol with imaging TOF-SIMS. J Microsc. 2004;215(2):156–61. 10.1111/j.0022-2720.2004.01374.x. 15315502

[35] Nygren H, Malmberg P, Kriegeskotte C, Arlinghaus HF. Bioimaging TOF‐SIMS: localization of cholesterol in rat kidney sections. FEBS Lett. 2004;566(1–3):291–3. 10.1016/j.febslet.2004.04.052. 15147911

[36] Altelaar AFM, Van Minnen J, Heeren RMA, Piersma SR. The influence of the cholesterol microenvironment in tissue sections on molecular ionization efficiencies and distributions in ToF-SIMS. Appl Surf Sci. 2006;252(19):6702–5. 10.1016/j.apsusc.2006.02.266.

[37] Nygren H, Börner K, Hagenhoff B, Malmberg P, Månsson J-E. Localization of cholesterol, phosphocholine and galactosylceramide in rat cerebellar cortex with imaging TOF-SIMS equipped with a bismuth cluster ion source. Biochimica et Biophysica Acta (BBA) - Molecular and Cell Biology of Lipids. 2005;1737(2–3):102–10. 10.1016/j.bbalip.2005.10.004. 16300993

[38] Shon HK, Kim SH, Yoon S, Shin CY, Lee TG. Molecular depth profiling on rat brain tissue sections prepared using different sampling methods. Biointerphases. 2018;13(3):03B411. 10.1116/1.5019611. 29495661

[39] Lagator M, Liu S, Mackay CL, Green F. Effects of vacuum exposure on sample stability for mass spectrometry analysis. Biointerphases. 2025;20(4):041011. 10.1116/6.0004632. 40856442

[40] Berrueta Razo I, Sheraz S, Henderson A, Lockyer NP, Vickerman JC. Mass spectrometric imaging of brain tissue by time-of-flight secondary ion mass spectrometry - how do polyatomic primary beams C 60 + , Ar 2000 + , water-doped Ar 2000 + and (H 2 O) 6000 + compare? Rapid Commun Mass Spectrom. 2015;29(20):1851–62. 10.1002/rcm.7285. PMC4989468 26411506

[41] Tian H, Wucher A, Winograd N. Reducing the matrix effect in organic cluster SIMS using dynamic reactive ionization. J Am Soc Mass Spectrom. 2016;27(12):2014–24. 10.1007/s13361-016-1492-z. PMC5218814 27659034

[42] Alnajeebi AM, Vickerman JC, Lockyer NP. The influence of polyatomic primary ion chemistry on matrix effects in secondary ion mass spectrometry analysis. Rapid Commun Mass Spectrom. 2018;32(22):1962–70. 10.1002/rcm.8265. 30133034

[43] Lagator M, Berrueta Razo I, Royle T, Lockyer NP. Sensitivity enhancement using chemically reactive gas cluster ion beams in secondary ion mass spectrometry (SIMS). Surf Interface Anal. 2022. 10.1002/sia.7054.

[44] Lagator M, Patel B, Sheraz S, Lockyer N. Reactive gas cluster ion beams for enhanced drug analysis by secondary ion mass spectrometry. Anal Chem. 2024;acs.analchem.4c02144. 10.1021/acs.analchem.4c02144. PMC11428081 39270000

[45] Yoon S, Lee TG. Biological tissue sample preparation for time-of-flight secondary ion mass spectrometry (ToF–SIMS) imaging. Nano Convergence. 2018;5(1):24. 10.1186/s40580-018-0157-y. PMC6153193 30467706

[46] Tian H, Sparvero LJ, Blenkinsopp P, Amoscato AA, Watkins SC, Bayır H, et al. Secondary‐ion mass spectrometry images cardiolipins and phosphatidylethanolamines at the subcellular level. Angew Chem Int Ed. 2019;58(10):3156–61. 10.1002/anie.201814256. PMC6622167 30680861

[47] Schindelin J, Arganda-Carreras I, Frise E, Kaynig V, Longair M, Pietzsch T, et al. Fiji: an open-source platform for biological-image analysis. Nat Methods. 2012;9(7):676–82. 10.1038/nmeth.2019 PMC3855844 22743772

[48] Seah MP. Universal equation for argon gas cluster sputtering yields. J Phys Chem C. 2013;117(24):12622–32. 10.1021/jp402684c.

[49] Wang S-K, Chang H-Y, Chu Y-H, Kao W-L, Wu C-Y, Lee Y-W, et al. Effect of Energy per Atom ( E / n ) on the Ar Gas Cluster Ion Beam (Ar-GCIB) and O 2 + Cosputter Process. Analyst. 2019;144(10):3323–33. 10.1039/C8AN02452A. 30968864

[50] Sheraz S, Tian H, Vickerman JC, Blenkinsopp P, Winograd N, Cumpson P. Enhanced ion yields using high energy water cluster beams for secondary ion mass spectrometry analysis and imaging. Anal Chem. 2019;91(14):9058–68. 10.1021/acs.analchem.9b01390. 31136149

[51] Lagator M, Sheraz S, Tian H, Green F, Lockyer N. Enhanced formation of multiply charged protein and peptide ions in SIMS using large water clusters. Anal Chem. 2025. 10.1021/acs.analchem.5c01508. 40420667

[52] Eckstein W, Garciá-Rosales C, Roth J, László J. Threshold energy for sputtering and its dependence on angle of incidence. Nucl Instrum Methods Phys Res Sect B Beam Interact Mater Atoms. 1993;83(1–2):95–109. 10.1016/0168-583X(93)95913-P.

[53] Lee JLS, Ninomiya S, Matsuo J, Gilmore IS, Seah MP, Shard AG. Organic depth profiling of a nanostructured delta layer reference material using large argon cluster ions. Anal Chem. 2010;82(1):98–105. 10.1021/ac901045q. 19957960

[54] Sano N, Bellew A, Blenkinsopp P. Comparing sputter rates, depth resolution, and ion yields for different gas cluster ion beams (GCIB): a practical guide to choosing the best GCIB for every application. J Vac Sci Technol A. 2023;41(5):053204. 10.1116/6.0002864.

[55] Taylor AJ, Graham DJ, Castner DG. Reconstructing accurate ToF-SIMS depth profiles for organic materials with differential sputter rates. Analyst. 2015;140(17):6005–14. 10.1039/C5AN00860C. PMC4532557 26185799

[56] Ekar J, Panjan P, Drev S, Kovač J. ToF-SIMS depth profiling of metal, metal oxide, and alloy multilayers in atmospheres of H 2 , C 2 H 2 , CO, and O 2. J Am Soc Mass Spectrom. 2022;33(1):31–44. 10.1021/jasms.1c00218. PMC8739835 34936371

[57] Delcorte A, Garrison BJ. Water nanodroplet impacts on surfaces: effect of the substrate nature. Nucl Instrum Methods Phys Res Sect B Beam Interact Mater Atoms. 2013;303:179–83. 10.1016/j.nimb.2012.10.029.

[58] Rzeznik L, Czerwinski B, Garrison BJ, Winograd N, Postawa Z. Microscopic insight into the sputtering of thin polystyrene films on Ag{111} induced by large and slow Ar clusters. J Phys Chem C. 2008;112(2):521–31. 10.1021/jp076667q.

[59] Delcorte A, Garrison BJ, Hamraoui K. Dynamics of molecular impacts on soft materials: from fullerenes to organic nanodrops. Anal Chem. 2009;81(16):6676–86. 10.1021/ac900746x. 20337378

[60] Breuker K, Knochenmuss R, Zhang J, Stortelder A, Zenobi R. Thermodynamic control of final ion distributions in MALDI: in-plume proton transfer reactions. Int J Mass Spectrom. 2003;226(1):211–22. 10.1016/S1387-3806(02)00965-X.

[61] Kański M, Hrabar S, Van Duin ACT, Postawa Z. Development of a charge-implicit ReaxFF for C/H/O systems. J Phys Chem Lett. 2022;13(2):628–33. 10.1021/acs.jpclett.1c03867. PMC8785188 35019649

[62] Tian H, Maciążek D, Postawa Z, Garrison BJ, Winograd N. C-O bond dissociation and induced chemical ionization using high energy (CO2)N+ gas cluster ion beam. J Am Soc Mass Spectrom. 2019;30(3):476–81. 10.1007/s13361-018-2102-z. PMC6417932 30430438

[63] Lee SJ, Hong A, Cho J, Choi CM, Baek JY, Eo JY, et al. Characteristics of a mixed-gas cluster ion beam for time-of-flight secondary ion mass spectrometry. Appl Surf Sci. 2022;572:151467. 10.1016/j.apsusc.2021.151467.

[64] Alnajeebi AM, Vickerman JC, Lockyer NP. Matrix effects in biological SIMS using cluster ion beams of different chemical composition. Biointerphases. 2016;11(2):02A317. 10.1116/1.4941009. 26825287

[65] Nezhad ZS, Salazar JP, Pryce RS, Munter LM, Chaurand P. Absolute quantification of cholesterol from thin tissue sections by silver-assisted laser desorption ionization mass spectrometry imaging. Anal Bioanal Chem. 2022;414(23):6947–54. 10.1007/s00216-022-04262-6. 35953724

[66] Piwowar AM, Fletcher JS, Kordys J, Lockyer NP, Winograd N, Vickerman JC. Effects of cryogenic sample analysis on molecular depth profiles with TOF-secondary ion mass spectrometry. Anal Chem. 2010;82(19):8291–9. 10.1021/ac101746h. 20836508

[67] Roddy TP, Cannon DM, Ostrowski SG, Ewing AG, Winograd N. Proton transfer in time-of-flight secondary ion mass spectrometry studies of frozen-hydrated dipalmitoylphosphatidylcholine. Anal Chem. 2003;75(16):4087–94. 10.1021/ac026353t. 14632121

[68] Conlan XA, Lockyer NP, Vickerman JC. <article-title update="added">Is proton cationization promoted by polyatomic primary ion bombardment during time‐of‐flight secondary ion mass spectrometry analysis of frozen aqueous solutions? Rapid Commun Mass Spectrom. 2006;20(8):1327–34. 10.1002/rcm.2446. 16555365

[69] Uzoni S, Zeidi S, Thoma J, Chatzikyriakos V, Neittaanmäki N, Fletcher JS. Secondary ion yield enhancement for lipids in frozen hydrated lymphoma tissue samples using water cluster SIMS. J Vac Sci Technol B. 2026;44(1):014002. 10.1116/6.0005143.

[70] Tian H, Sparvero LJ, Anthonymuthu TS, Sun W-Y, Amoscato AA, He R-R, et al. <article-title update="added"> Successive high-resolution (H _2_ O) _*n*_ -GCIB and C _60_ -SIMS imaging integrates multi-omics in different cell types in breast cancer tissue. Anal Chem. 2021;93(23):8143–51. 10.1021/acs.analchem.0c05311. PMC8209780 34075742

[71] Zhang J, Brown J, Scurr DJ, Bullen A, MacLellan-Gibson K, Williams P, et al. Cryo-OrbiSIMS for 3D molecular imaging of a bacterial biofilm in its native state. Anal Chem. 2020;92(13):9008–15. 10.1021/acs.analchem.0c01125. 32460495

[72] Ostrowski SG, Kurczy ME, Roddy TP, Winograd N, Ewing AG. Secondary ion MS imaging to relatively quantify cholesterol in the membranes of individual cells from differentially treated populations. Anal Chem. 2007;79(10):3554–60. 10.1021/ac061825f. PMC1905852 17428032

