# Supplementary material for: Improvements in high-yield detection and localisation of cholesterol in murine brains using water cluster beam secondary ion mass spectrometry
Source: Anal Bioanal Chem. 2026 Mar 26;418(20):6691–704. doi: 10.1007/s00216-026-06448-8 (PMC13619783; doi:10.1007/s00216-026-06448-8)
Supplement: Supplementary file 1 [file 216_2026_6448_MOESM1_ESM.docx]

**Supplemental Information**

**Improvements in high-yield detection and localisation of cholesterol in murine brains using water cluster beam secondary ion mass spectrometry**

Matija Lagator^1,2^, Irma Berrueta Razo^1,3^, Sadia Sheraz^1^, Nicholas P. Lockyer^1^*

^1^ Department of Chemistry, Photon Science Institute, The University of Manchester, Oxford Road, Manchester, M13 9PL, United Kingdom

2 Rosalind Franklin Institute, Building R113 Rutherford Appleton Laboratory, Harwell Campus, Didcot, Oxfordshire, OX11 0QX, United Kingdom

^3^Medicines Discovery Catapult, 35 Mereside Alderley Park, Macclesfield SK10 4ZF, UK

*Nick.Lockyer@manchester.ac.uk

**Table S1.** Showing experimental parameters for cryogenic imaging of rat brains.

| Primary Ion Type | GCIB | GCIB | GCIB | GCIB |
| --- | --- | --- | --- | --- |
| Cluster Type | Ar/CO_2_ | Ar/CO_2_ | H_2_O | H_2_O |
| Ion Mode | Positive | Negative | Positive | Negative |
| Cluster Size | 6000 | 6000 | 15000 | 15000 |
| Current (pA) | 45 | 45 | 33 | 33 |
| Raster Area (μm^2^) | 450 | 450 | 450 | 450 |
| Pixels in Raster Area | 64 × 64 | 64 × 64 | 64 × 64 | 64 × 64 |
| Number of Tiles (x,y) | 4 × 2 | 4 × 2 | 4 × 2 | 4 × 2 |
| Ion Dose (ions/cm^2^) | 1.09 × 10^13^ | 1.09 × 10^13^ | 1.08 × 10^13^ | 1.08 × 10^13^ |

Table S2. Showing the cluster size and the measured time of flight used to derive the cluster size. The full width at half maximum (FWHM), reported in both microseconds and as a percentage, represents the range of time-of-flight values detected during cluster size measurements. Data are shown for experiments performed on cholesterol standards using (ArCO₂)ₙ and (H₂O)ₙ clusters in both positive and negative ion modes.

A

B

D

E

C

F

**Figure S1.** ToF-SIMS spectra of mouse brain samples collected with varying primary ions: A and D – (H_2_O)_n_; B and E – (ArCO_2_)_n_; C and F – C_60_. Top row shows positive ion mode spectra while the bottom row shows negative ion mode spectra. All experiments were done on dry brain sections at room temperature.

**Figure S2.** ToF-SIMS spectrum of mouse brains in positive mode, showing the intensities with three different ion beams. Bottom panel is a zoomed in area of the top panel. All experiments were done on dry brain sections at room temperature.

**Figure S3.** ToF-SIMS spectrum of mouse brains in negative mode, showing the intensities with three different ion beams. Bottom panel is a zoomed in area of the top panel. All experiments were done on dry brain sections at room temperature.

**Figure S4.** Comparison of positive and negative mode lipid ToF-SIMS signal yields from rat brain under cryogenic conditions. Ratio of signal intensity at corresponding *m/z* value and the total ion intensity is shown. Data represents signals from the whole brain area that had been analysed. Note that the *m/z* 184 and *m/z* 283 data are plotted separately due to large discrepancy in signal between these peaks and the rest of the *m/z* values measured. Additionally, the *m/z* 55 [(H_2_O)_3_+H]^+^ and *m/z* 53 [(H_2_O)_3_-H]^-^ peaks are representative of the detected levels of water on the samples.

**Figure S5.** Change in cholesterol concentration (on Si wafer) over time. Signal is stable for the 25 areas that were analysed (consecutive analysis regions on the same wafer that had been coated with cholesterol standard). The periodic decreases in signal are likely caused by the edge effect, meaning that those analysis areas were closer to the edge of the wafer and were therefore representative of a thinner layer of cholesterol. Experiment was done at room temperature.

**Figure S6.** Cholesterol standard signal comparison on day 1 and day 4 in vacuum. The signal increased slightly but this is potentially due to differences in experimental variables (slight changes in temperature, ion dose, etc.). Overall graphs S5 and S6 show that the “vacuum effect” (sample sublimation) does not significantly affect cholesterol standards. Experiment was done at room temperature.

**Figure S7.** Showing the fragmentation ratio and the dimerisation ratio of cholesterol standards on Si wafers. A - fragmentation in positive ion mode (*m/z* 369.35/161.13); B – fragmentation in negative ion mode (*m/z* 385.38/283.28); C – dimerisation in negative ion mode (*m/z* 771.76/385.38). Experiment was done at room temperature.

**Figure S8.** - Showing the fragmentation ratio of cholesterol standards on Si wafers in positive ion mode (*m/z* 369.35/161.13); Both the (ArCO_2_)_n_ and (H_2_O)_n_ clusters were compared at *E/m* of 0.35 eV/amu. Experiment was done at room temperature.

**Figure S9.** Relative spot size measurements of imaged brain sections (Figure 3). Line scans of intensity profiles were used to determine the spatial resolution by applying the 20–80% criterion. Ten independent line scans were performed, averaged, and normalised to the spot size measured using (H_2_O)_n_ clusters. The resulting values represent relative spot sizes compared to the (H_2_O)_n_ cluster reference. A) Positive ion mode; B) Negative ion mode. All experiments were done on dry brain sections at room temperature.
